# Temporal trends, patient characteristics and hospital volume outcomes after transcoronary ablation of septal hypertrophy and septal myectomy for treatment of obstructive hypertrophic cardiomyopathy

**DOI:** 10.1007/s00392-025-02773-7

**Published:** 2025-10-09

**Authors:** Peter Moritz Becher, Fabian J. Brunner, Jessica Weimann, Marko Remmel, Benedikt Schrage, Monica Patten, Christian Detter, Hermann Reichenspurner, Paulus Kirchhof, Stefan Blankenberg, Moritz Seiffert

**Affiliations:** 1 Department of Cardiology, University Heart and Vascular Center Hamburg, Martinistrasse 52, 20246 Hamburg, Germany; 2 German Center for Cardiovascular Research (DZHK), Partner site Hamburg/Kiel/Lübeck, Hamburg, Germany; 3 Center for Population Health Innovation (POINT), University Heart and Vascular Center Hamburg, University Medical Center Hamburg-Eppendorf, Hamburg, Germany; 4 Department of Cardiovascular Surgery, University Heart & Vascular Center Hamburg, Hamburg, Germany; 5 Institute of Cardiovascular Sciences, University of Birmingham, Birmingham, UK; 6 Department of Cardiology and Angiology, BG University Hospital Bergmannsheil, Ruhr-University Bochum, Bochum, Germany

**Keywords:** Hypertrophic obstructive cardiomyopathy, Septal reduction therapy, Alcohol septal ablation, Septal myectomy, Hospital volume, Outcome

## Abstract

**Background:**

Hypertrophic obstructive cardiomyopathy (HOCM) is a hereditary cardiac disease associated with poor prognosis. Data on septal reduction therapy (SRT), including transcoronary ablation of septal hypertrophy (TASH) and surgical myectomy (SM), are limited. We aimed to evaluate temporal trends, patient characteristics, in-hospital outcomes, and the influence of institutional procedural volumes in a nationwide cohort.

**Methods:**

We used nationwide administrative data from the German Federal Bureau of Statistics to identify HOCM patients undergoing TASH or SM between 2006 and 2019. Primary outcomes were in-hospital mortality and pacemaker implantation. Multivariable logistic regression assessed associations with institutional procedure volume.

**Results:**

Among 8,514 SRT procedures, 5,293 (62.2%) were TASH and 3,221 (37.8%) SM. Annual volumes increased for both procedures between 2006 and 2019. SM patients were older (67.4 vs. 60.2 years), more often male, and had a higher comorbidity burden. Of all centers, 49.3% performed ≤ 20 SM per year, and 76.3% performed ≤ 20 TASH per year. Mitral surgery occurred in 36.1% of SM cases. In-hospital mortality was higher for SM than TASH (6.9% vs. 0.8%), while pacemaker implantation was more frequent with TASH (19.3% vs. 13.1%). Risk of in-hospital mortality after SM was higher in low-volume centers than in large centers (adjusted odds ratio 2.64; 95% confidence interval, 1.49–4.66).

**Conclusion:**

SM was performed in older, more comorbid patiens and frequently included concomitant valve surgery. Higher in-hospital mortality at low-volume centers may partially reflect surgical complexity or case mix rather than volume alone. Our findings underscore the need for individualized referral strategies to experienced centers.

**Graphical Abstract:**

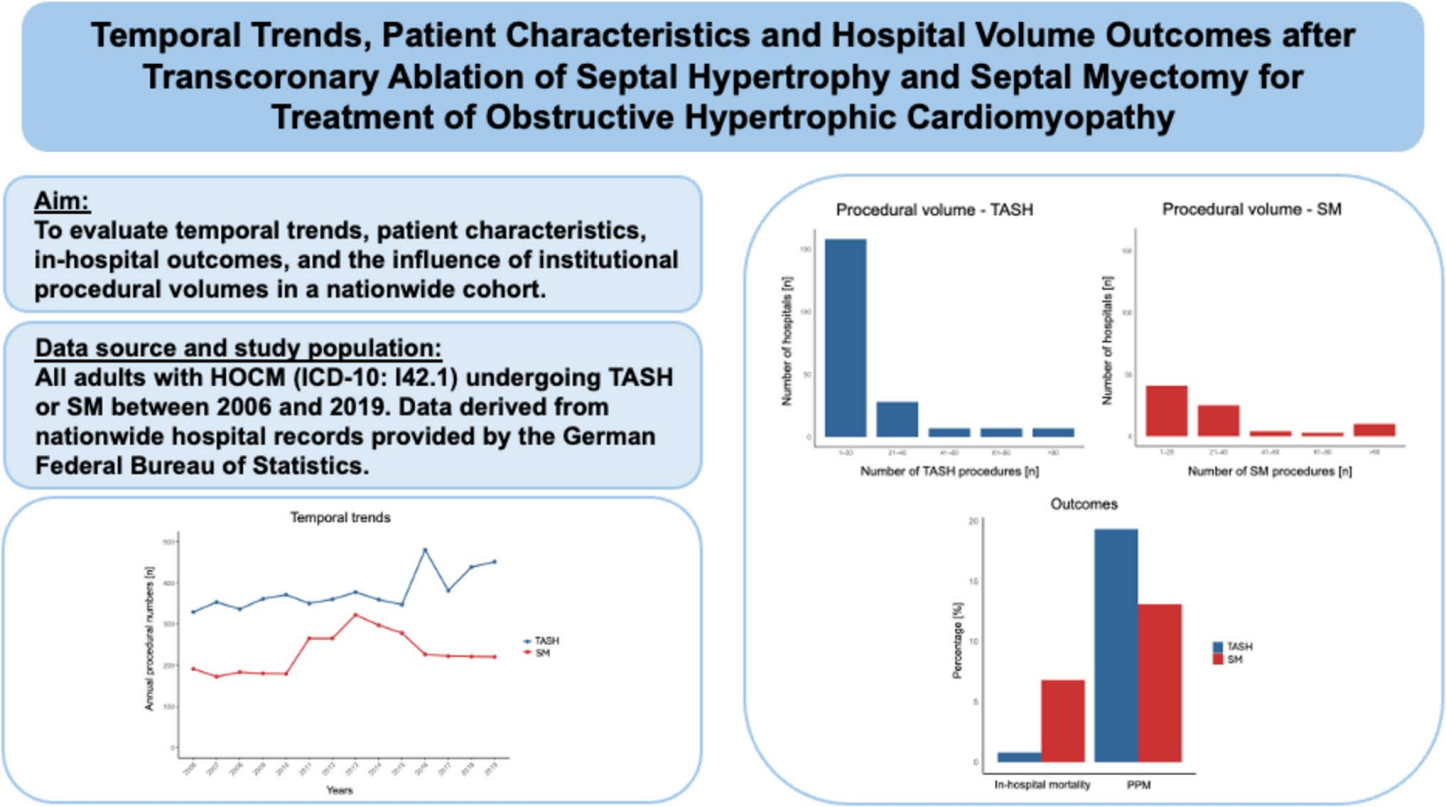

**Supplementary information:**

The online version contains supplementary material available at 10.1007/s00392-025-02773-7.

## Introduction

Hypertrophic cardiomyopathy (HCM) is an inherited cardiac condition that affects approximately 1 in 500 individuals in the general population [1–3]. Among those diagnosed with HCM, up to 70% develop some degree of left ventricular outflow tract (LVOT) obstruction, a condition classified as hypertrophic obstructive cardiomyopathy (HOCM) [4]. HOCM associated with severe symptoms, such as dyspnea, exertional syncope, and an elevated risk of sudden cardiac death [5, 6]. Septal reduction therapies (SRT), including transcoronary ablation of septal hypertrophy (TASH) and surgical septal myectomy (SM), are established treatments for patients with severe drug-refractory symptoms [7, 8]. While TASH is often preferred for patients with high surgical risk, SM remains the gold standard in those who are suitable surgical candidates, according to recent ESC and ACC/AHA guidelines [6, 9, 10]. These guidelines emphasize that SRT should be performed at centers with expertise, as procedural volume has been closely linked to outcomes [6, 9, 10].

Myosin inhibitors represent a novel pharmacological treatment for symptomatic relief in HOCM. However, these agents were not available during the study period and do not replace SRT in patients with advanced, drug-refractory LVOT obstruction [11, 12].

Previous studies indicate that low-volume centers are associated with higher complication rates, including increased in-hospital mortality and a higher need for permanent pacemaker implantation, particularly for SM [13–15]. However, contemporary large-scale data, especially from regions outside the U.S., remain scarce.

This study aimed to examine temporal trends in a nationwide cohort of patients with HOCM and hospitalized for SRT in Germany from 2006 to 2019, addressing: 1) utilization patterns of TASH vs. SM; 2) patient characteristics; 3) key clinical outcomes, including in-hospital mortality and pacemaker implantation rates; and 4) the association between institutional procedural volumes and clinical outcomes.

## Materials and methods

### Data source

This retrospective cohort study used anonymized nationwide inpatient data from the German Federal Bureau of Statistics, encompassing all hospitalizations in Germany from January 1, 2006, to December 31, 2019 (Source: RDC of the Federal Statistical Office and Statistical Offices of the Länder, DRG-Statistik 2006–2019, own calculations). Diagnoses and procedures were identified using the International Classification of Diseases, 10th Revision, German Modification (ICD-10-GM), and the German Operation and Procedure Classification System (OPS), respectively.

### Study population

All adult patients with a primary or secondary diagnosis of HOCM, defined by ICD-10-GM code I42.1, who underwent either TASH (OPS code 5–377.70) or SM (OPS code 5–350.4) were included. Patients who received both TASH and SM during the same hospitalization were excluded from the analysis.

### Study design

This study aimed to assess temporal trends, patient profiles, in-hospital outcomes, and the association between institutional procedural volume and clinical outcomes in patients undergoing SRT. Annual hospital procedural volumes were calculated for each institution. Hospitals were stratified into volume groups based on the sum across annualized procedural frequency for TASH and SM. To investigate the association between procedural volume and outcomes in a clinically meaningful and interpretable manner, we defined hospital volume categories according to absolute thresholds. For both TASH and SM, we categorized centers based on annual case volume into low volume (1 to 20 procedures), intermediate volume (21 to 40 procedures), moderate-high volume (41 to 60 procedures), high volume (61 to 80 procedures), and very high volume (more than 80 procedures). These cut-offs were chosen based on visual inspection of the procedural volume distribution across centers, ensuring clear stratification while preserving sufficient sample size within each category. The thresholds were designed to reflect real-world differences in institutional experience and align with existing recommendations on center expertise. Categorization based on absolute numbers may also facilitate interpretability for clinicians and policymakers.

In addition to this absolute categorization, hospitals were also stratified into tertiles based on annual procedural volume for TASH and SM. For TASH, tertiles were defined as fewer than 2 procedures, 2 to 13 procedures, and 13 or more procedures. For SM, tertiles were defined as fewer than 13 procedures, 13 to 30 procedures, and 30 or more procedures. These tertile thresholds were derived from the empirical distribution of procedural volumes in the dataset to allow balanced group sizes and robust statistical comparisons. Tertile-based classification supports inferential modeling and ensures comparability across institutions with varying patient volumes.

### Outcomes

The primary outcomes were in-hospital mortality and need for permanent pacemaker implantation during index hospitalization. Secondary outcomes included mechanical circulatory support, acute renal failure, bleeding complications, and stroke.

### Statistical analysis

Baseline characteristics were summarized using means and standard deviations for continuous variables, and proportions for categorical variables. Group differences were assessed using the chi-square test for categorical variables and the Student’s t-test for continuous variables, depending on data distribution.

To evaluate the influence of hospital procedural volume on clinical outcomes, multivariable logistic regression models were fitted. These models were adjusted for relevant clinical and demographic features, including age, sex (female), anemia, heart failure, chronic obstructive pulmonary disease (COPD), diabetes mellitus, coagulopathies, arterial hypertension, liver disease, obesity, peripheral artery disease, chronic kidney disease, and history of bypass graft or valve surgery. In addition, institutional procedural volume was included in the final models as a categorical variable based on tertile classification for TASH and SM, respectively. This enabled adjustment for institutional experience in addition to patient-level characteristics. The results are presented as adjusted odds ratios (aORs) with corresponding 95% confidence intervals (CIs).

All statistical analyses were conducted using R version 4.0.3. Statistical significance was defined as a two-tailed p-value of less than 0.05.

## Results

### Utilization patterns and temporal trends

**Fig. 1:**
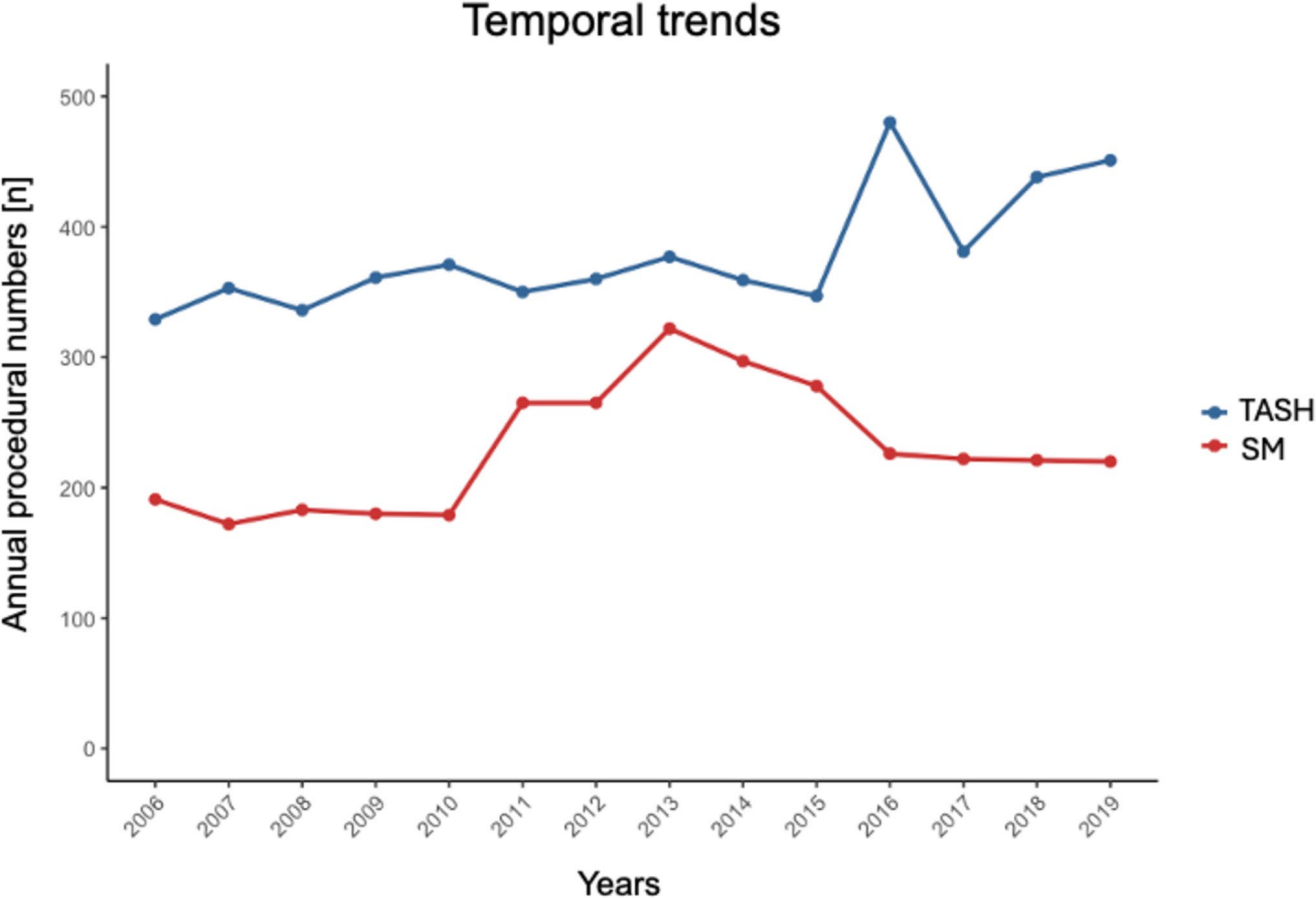
Temporal trends of transcoronary ablation of septal hypertrophy (TASH) and septal myectomy (SM) performed procedures over time

**Fig. 2:**
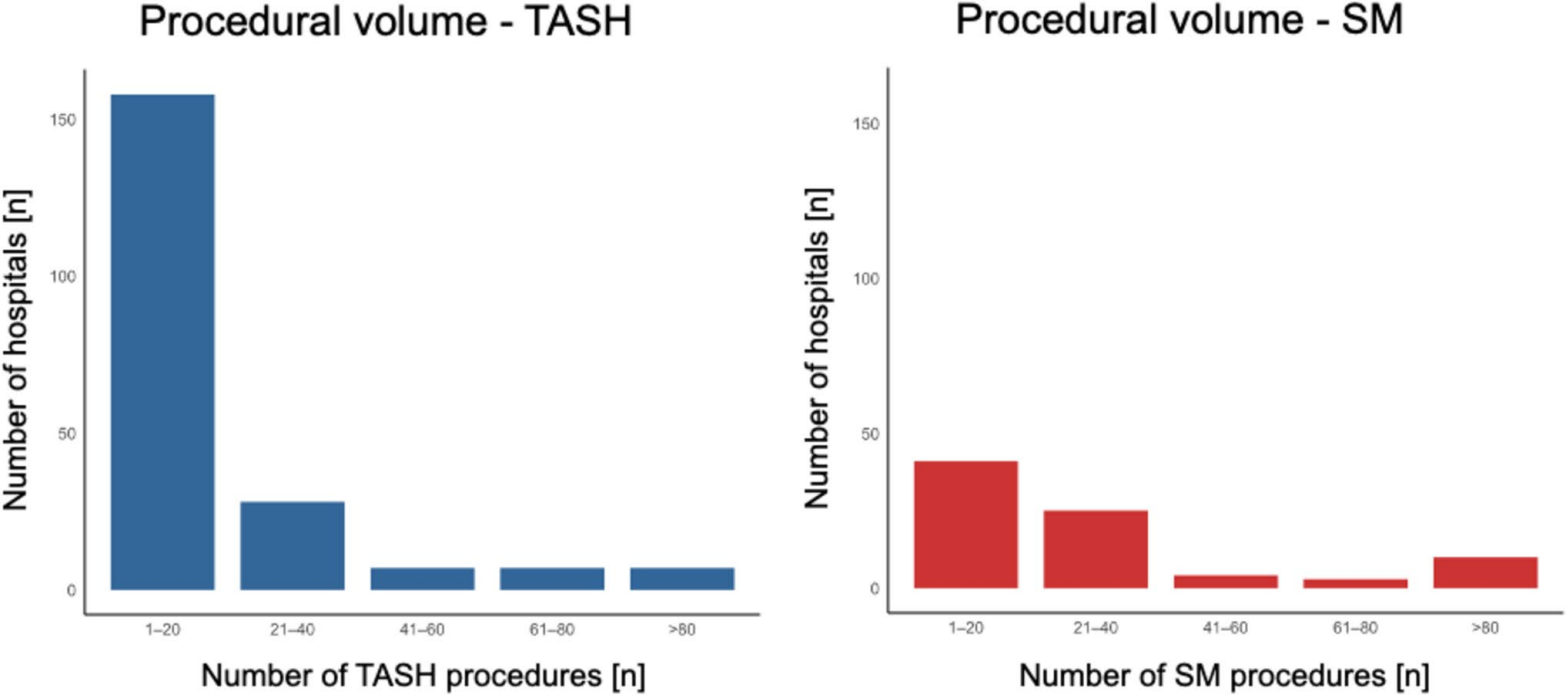
Number of transcoronary ablation of septal hypertrophy (TASH) and septal myectomy (SM) procedures performed across sites

Between 2006 and 2019, 8,514 patients with HOCM underwent septal reduction therapy (SRT) in Germany. TASH was performed in 5,293 patients (62.2%), while 3,221 patients (37.8%) underwent SM. Procedural volumes for both interventions increased over time. TASH procedures increased from 329 in 2006 to 451 in 2019, whereas SM procedures increased from 191 to 220 during the same period **(**Fig. 1**)**. Among centers performing SM, 49.3% (41 of 83) conducted ≤ 20 procedures **(**Fig. 2**)**. For TASH, 76.3% of centers (158 of 207) performed ≤ 20 procedures.

### Patient characteristics

Patients undergoing SM were older (mean age 67.4 vs. 60.2 years for TASH) and had a higher burden of comorbidities compared with TASH, including atrial fibrillation (46.1% vs. 18.4%), diabetes mellitus (18.5% vs. 11.8%), heart failure (12.2% vs. 3.8%), and chronic kidney disease (14.3% vs. 9.2%) **(**Table 1**)**. A higher proportion of TASH patients were women (56.4%) compared with SM patients (49.9%). The mean length of hospital stay was longer for SM patients (18.1 ± 14.2 days) compared with TASH patients (10.5 ± 7.0 days). Concomitant mitral valve surgery was performed in 36.1% of SM patients compared with only 0.08% of TASH patients (*p* < 0.001) **(**Table 1**)**.

**Table 1:** Baseline characteristics, treatments, and outcomes of patients undergoing septal reduction therapy (SRT)

Variables	All (*n* = 8514)	SM (*n* = 3221)	TASH (*n* = 5293)	*p*-value
Demographics				
Age (years, SD)	62.9 ± 14.0	67.4 ± 12.7	60.2 ± 14.1	< 0.001
Female (%)	4461 (52.4)	1818 (56.4)	2643 (49.9)	< 0.001
Length of hospital stay (days)	13.4 ± 11.0	18.2 ± 14.2	10.6 ± 7.1	< 0.001
Comorbidities				
Arterial hypertension (%)	1571 (18.5)	551 (17.1)	1020 (19.3)	0.014
Hyperlipoproteinemia (%)	3379 (39.7)	1373 (42.6)	2006 (37.9)	< 0.001
Diabetes mellitus (%)	1223 (14.4)	597 (18.5)	626 (11.8)	< 0.001
Atrial fibrillation (%)	2462 (28.9)	1485 (46.1)	977 (18.5)	< 0.001
Obesity (%)	1224 (14.4)	575 (17.9)	649 (12.3)	< 0.001
COPD (%)	61 (0.7)	52 (1.6)	9 (0.2)	< 0.001
Anemia (%)	2373 (27.9)	2194 (68.1)	179 (3.4)	< 0.001
Pulmonary hypertension (%)	157 (1.8)	56 (1.7)	101 (1.9)	0.63
Chronic kidney disease (%)	950 (11.2)	462 (14.3)	488 (9.2)	< 0.001
Heart failure (%)	603 (7.1)	400 (12.4)	203 (3.8)	< 0.001
Stroke (%)	322 (3.8)	289 (9.0)	33 (0.6)	< 0.001
Liver disease (%)	228 (2.7)	175 (5.4)	53 (1.0)	< 0.001
Coagulopathy (%)	2320 (27.3)	1506 (46.8)	814 (15.4)	< 0.001
Invasive ventilation (%)	909 (10.7)	817 (25.4)	92 (1.7)	< 0.001
Non-invasive ventilation (%)	407 (4.8)	346 (10.7)	61 (1.2)	< 0.001
Dialysis (%)	460 (5.4)	428 (13.3)	32 (0.6)	< 0.001
History of MI (%)	245 (2.9)	153 (4.8)	92 (1.7)	< 0.001
History of CABG (%)	82 (1.0)	65 (2.0)	17 (0.3)	< 0.001
PAD (%)	3588 (42.1)	1500 (46.6)	2088 (39.5)	< 0.001
Coronary intervention (%)	5323 (62.5)	30 (0.9)	5293 (100)	< 0.001
Coronary angiogram (%)	5349 (62.8)	748 (23.2)	4601 (86.9)	< 0.001
Mitral valve surgery (%)	1168 (13.7)	1164 (36.1)	4 (0.1%)	< 0.001
Outcomes				
Acute renal failure (%)	120 (1.4)	46 (1.4)	74 (1.4)	0.985
Bleeding/transfusion (%)	2522 (29.6)	2339 (72.6)	183 (3.5)	< 0.001
Mechanical circulatory support (%)	129 (1.5)	114 (3.5)	15 (0.3)	< 0.001
Pacemaker implantation (%)	1442 (16.9)	421 (13.1)	1021 (19.3)	< 0.001
In-hospital mortality (%)	262 (3.1)	221 (6.9)	41 (0.8)	< 0.001

*CABG* Coronary artery bypass graft, *COPD* chronic obstructive pulmonary disease, *MI* myocardial infarction, *PAD* peripheral artery disease, *SD* standard deviation, *SRT* septal reduction therapy, *SM* surgical myectomy, *TASH* transcoronary ablation of septal hypertrophy

Between 2006 and 2019, patient characteristics for SRT evolved differently for TASH and SM. For TASH, mean age increased from 58.6 to 62.3 years (*p* < 0.001), with stable female representation (~ 50%) and a reduction in hospital length of stay from 12.4 to 9.0 days. For SM, mean age declined slightly from 68.3 to 65.6 years (*p* = 0.003), with a reduction in the proportion of females from 62.3% to 45.9% (*p* = 0.006) and a shorter hospital stay from 22.8 to 16.2 days (*p* < 0.001) (Supplemental Tables S1 and S2).

Patients treated at low-volume centers for both TASH and SM were older and had a higher comorbidity burden compared with those treated at high-volume centers (Supplemental Tables S3 and S4). In TASH, those at low-volume hospitals had more atrial fibrillation (39.1% vs. 17.9%), diabetes (21.7% vs. 11.6%), and chronic kidney disease (19.6% vs. 8.9%), along with longer hospital stays (12.9 vs. 10.5 days; all *p* < 0.05). Similarly, SM patients in low-volume centers had longer hospital stays (21.9 vs. 18.0 days), more atrial fibrillation (49.7% vs. 40.4%), and higher in-hospital mortality (10.8% vs. 6.3%; *p* = 0.037), compared with those treated at high-volume institutions (Supplemental Tables S3 and S4).

### Clinical outcomes

**Fig. 3:**
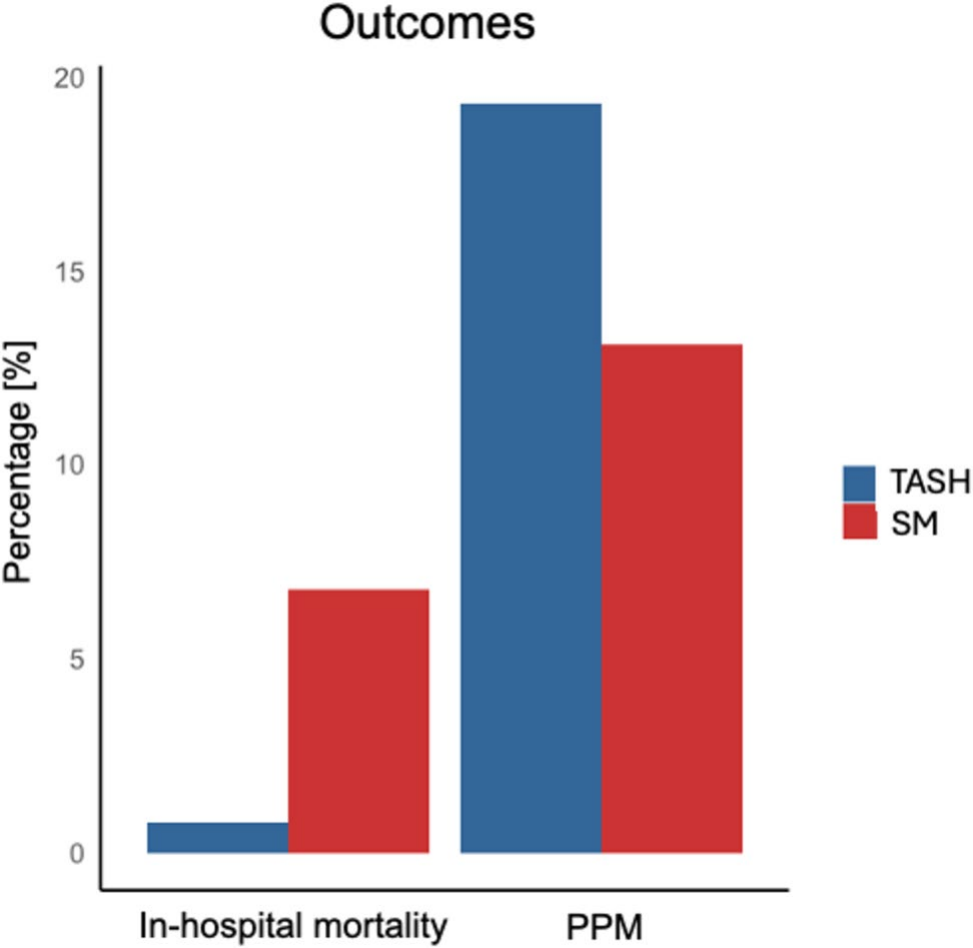
In-hospital outcomes in patients undergoing transcoronary ablation of septal hypertrophy (TASH) and septal myectomy (SM) procedures. permanent pacemaker (PPM)

In-hospital mortality was significantly higher in the SM group (6.9%) compared with the TASH group (0.8%) **(**Table 1,Fig. 3**)**. Pacemaker implantation was more frequently required in TASH patients (19.3% vs. 13.1% for SM, *p* < 0.001) **(**Table 1,Fig. 3). Both interventions had low rates of other complications. SM was associated with a higher frequency of invasive procedures compared with TASH, including advanced postoperative management requiring advanced mechanical circulatory support (3.5% vs. 0.3%) or respiratory support (invasive ventilation: 25.4% vs. 1.7%; non-invasive ventilation: 10.7% vs. 1.2%) **(**Table 1**)**.

### Association between institutional procedural volumes and clinical outcomes

In multivariable logistic regression analysis, SM performed at low-volume centers was independently associated with an increased risk of in-hospital mortality (adjusted odds ratio [aOR], 2.64; 95% CI, 1.49–4.66, reference third tertile). No significant associations were observed between institutional SM volume and the incidence of pacemaker implantation, mechanical circulatory support, stroke or acute renal failure **(**Table 2**)**.

**Table 2:** Unadjusted and adjusted associations between hospital volume and outcomes after septal alcohol ablation

Outcomes	Unadjusted OR (95% CI)	*p*-value	Adjusted OR (95% CI)	*p*-value
In-hospital mortality				
Second vs. third tertile	0.64 (0.15–2.65)	0.54	0.41 (0.09–1.84)	0.25
First vs. third tertile	2.81 (0.38–20.93)	0.31	1.66 (0.20–13.68)	0.64
Need for PPM				
Second vs. third tertile	0.88 (0.67–1.15)	0.35	0.84 (0.64–1.10)	0.22
First vs. third tertile	0.74 (0.33–1.66)	0.47	0.63 (0.28–1.42)	0.27
Stroke				
Second vs. third tertile	2.83 (1.16–6.92)	0.02	2.53 (1.01–6.35)	0.05
First vs. third tertile	4.12 (0.55–31.03)	0.17	2.56 (0.32–20.31)	0.37
MCS				
Second vs. third tertile	1.87 (0.42–8.33)	0.41	1.36 (0.26–7.01)	0.72
First vs. third tertile	-	-	-	-
Acute renal failure				
Second vs. third tertile	0.17 (0.02–1.24)	0.08	0.15 (0.02–1.10)	0.06
First vs. third tertile	4.76 (1.44–15.72)	0.01	3.34 (0.96–11.64)	0.06
Bleeding or transfusion				
Second vs. third tertile	1.38 (0.84–2.28)	0.20	1.08 (0.49–2.40)	0.85
First vs. third tertile	3.57 (1.39–9.16)	0.01	1.62 (0.38–6.96)	0.52

Adjusted models included age, sex, relevant comorbidities, and institutional procedural volume categorized by tertiles

*CI* Confidence interval, *MCS* mechanical circulatory support, *OR* odds ratio, *PPM* permanent pacemaker

Procedural volume showed no significant association with outcomes for TASH, including in-hospital mortality, need of pacemaker implantation, need of mechanical circulatory support, and acute renal failure **(**Table 3**)**.

**Table 3:** Unadjusted and adjusted associations between hospital volume and outcomes after septal myectomy

Outcomes	Unadjusted OR (95% CI)	*p*-value	Adjusted OR (95% CI)	*p*-value
In-hospital mortality				
Second vs. third tertile	1.31 (0.93–1.84)	0.12	1.45 (0.99–2.13)	0.06
First vs. third tertile	1.79 (1.07–3.00)	0.03	2.64 (1.49–4.66)	< 0.001
Need for PPM				
Second vs. third tertile	1.09 (0.83–1.42)	0.54	1.10 (0.84–1.44)	0.50
First vs. third tertile	1.20 (0.77–1.87)	0.42	1.27 (0.82–1.98)	0.29
Stroke				
Second vs. third tertile	0.68 (0.47–0.96)	0.03	0.74 (0.52–1.06)	0.10
First vs. third tertile	0.73 (0.40–1.33)	0.30	0.80 (0.44–1.47)	0.47
MCS				
Second vs. third tertile	0.47 (0.25–0.89)	0.02	0.44 (0.23–0.85)	0.01
First vs. third tertile	0.44 (0.14–1.39)	0.16	0.56 (0.17–1.84)	0.34
Acute renal failure				
Second vs. third tertile	1.80 (0.94–3.45)	0.08	2.06 (1.06–3.99)	0.985
First vs. third tertile	0.46 (0.06–3.40)	0.45	0.54 (0.07–4.02)	0.55
Bleeding or transfusion				
Second vs. third tertile	0.90 (0.74–1.10)	0.32	1.17 (0.85–1.62)	0.34
First vs. third tertile	1.00 (0.71–1.43)	0.98	1.30 (0.75–2.25)	0.34

Adjusted models included age, sex, relevant comorbidities, and institutional procedural volume categorized by tertiles

*CI* Confidence interval, *MCS* mechanical circulatory support, *OR* odds ratio, *PPM* permanent pacemaker

## Discussion

This nationwide study provides an institutional-level contemporary overview of SRT for HOCM in Germany. The main findings of our study were:

1. TASH is more commonly used (62.2%) than SM (37.8%);
2. the procedural volume of both TASH and SM increased steadily from 2006 to 2019, with TASH accounting for approximately two-thirds of all procedures;
3. nearly half of SM centers (49.3%) and over three-quarters of TASH centers (76.3%) performed 20 or fewer procedures, indicating limited centralization of SRT in Germany;
4. SM patients were older, had a higher comorbidity burden, and required concomitant mitral valve surgery in more than one-third of cases, reflecting increased procedural complexity;
5. SM was associated with higher in-hospital mortality (7%) compared with TASH (1%), while TASH had a higher rate of permanent pacemaker implantation (19% vs. 13%); and.
6. low institutional volume was independently associated with increased in-hospital mortality after SM, although this may partly reflect case mix and surgical complexity, highlighting the need for centralization of SM at high-volume centers.

Our findings show that both SM and TASH were frequently performed at low-volume centers, with nearly half of SM centers (49.3%) and over three quarters of TASH centers (76.3%) conducting 20 or fewer procedures. This distribution highlights a limited centralization of care for septal reduction therapies in Germany. Between 2006 and 2019, the annual number of TASH and SM procedures increased steadily, reflecting the growing recognition of SRT as a cornerstone in the management of symptomatic, drug-refractory HOCM [6, 9, 10]. Although myosin inhibitors are now recommended as first-line therapy in selected patients with HOCM, they do not replace SRT in those with advanced or complex disease [6, 9]. Their use is limited in cases with reduced ejection fraction or relevant drug interactions, and long-term safety data are lacking. SM remains the preferred option in patients with structural abnormalities, reinforcing the continued role of both SM and TASH and the importance of maintaining expertise at specialized centers.

In this study, TASH was the more frequently performed procedure, accounting for 62.2% of all SRT cases, compared with 37.8% for SM. This preference likely reflects the minimally invasive nature of TASH, its shorter recovery time, and its suitability for patients with high surgical risk.

Distinct differences in patient characteristics were observed between TASH and SM groups, highlighting the tailored application of these procedures. Patients undergoing SM were older (mean age 67.4 vs. 60.2 years for TASH) and had a higher comorbidity burden, including atrial fibrillation, diabetes, heart failure, and chronic kidney disease. This reflects the fact that SM is often reserved for patients with more severe disease or anatomical complexities unsuitable for TASH [16]. In contrast, TASH patients had a higher proportion of females (56.4% vs. 49.9% for SM), suggesting potential sex-based differences in referral patterns or anatomical suitability for the procedure [17]. These differences emphasize the importance of individualized treatment planning in SRT [6, 9].

Over time, patient profiles for SRT have evolved, reflecting broader trends in HOCM management. In TASH, the mean patient age increased from 58.6 to 62.3 years, suggesting its growing use in older populations, supported by evidence of its safety and efficacy in the elderly [18–20]. Hospital stays decreased from 12.4 to 9.0 days, reflecting procedural standardization and improved care. Decreases in arterial hypertension and rising obesity rates align with population health trends [21]. For SM, the slight decline in mean age (68.3 to 65.6 years) and reduced proportion of female patients (62.3% to 45.9%) suggest a shift toward younger, anatomically complex cases. Shorter hospital stays (22.8 to 16.2 days) point to advances in surgical techniques, while increases in chronic kidney disease and anemia highlight growing case complexity [22]. These trends emphasize the adaptation of SRT to evolving patient needs, underscoring the importance of individualized approaches and specialized care centers [6, 9, 10].

Our study revealed significant disparities in in-hospital outcomes between TASH and SM. SM was associated with higher in-hospital mortality (7%) compared with TASH (1%). This likely reflects the higher procedural complexity and higher comorbidity burden in SM patients, including more frequent chronic kidney disease, COPD, diabetes, obesity, and heart failure. These differences limit direct comparability between groups and underline the importance of risk adjustment in future analyses. On the other hand, TASH patients were more likely to require permanent pacemaker implantation (19.3% vs. 13.1% for SM). These outcomes highlight the trade-offs between the procedures: while TASH offers a safer short-term profile, it may lead to device-related morbidity due to conduction system disruption. SM remains the gold standard for patients with advanced LVOT obstruction, particularly when concomitant surgical procedures are necessary [6, 9, 10]. Notably, similar findings on mortality differences between TASH and SM were reported in a study by Kim et al. which observed a significantly lower mortality rate in TASH patients compared with SM patients [13]. In a study by Liebregts et al., the need for permanent pacemaker implantation after TASH was age-dependent, with rates of 8% in patients ≤ 50 years and 16% in those ≥ 65 years [23]. In our study, TASH patients had a higher permanent pacemaker implantation rate (19.3%) compared with SM patients (13.1%). These findings align with variations reported across studies, which may reflect differences in patient selection, procedural techniques, and institutional expertise, highlighting the importance of individualized treatment planning [24].

The association between institutional procedural volume and clinical outcomes was particularly evident for SM. Treatment at low-volume centers was independently associated with a higher risk of in-hospital mortality compared with high-volume centers (aOR, 2.64; 95% CI, 1.49–4.66). This finding underscores the technical complexity of SM and the importance of experience and expertise in achieving optimal outcomes. In contrast, procedural volume did not significantly impact TASH outcomes, likely due to the standardized and less invasive nature of the procedure. However, further differentiation between isolated SM and combined procedures (e.g., SM and mitral valve surgery or coronary artery bypass graft) was not feasible due to data protection restrictions and small subgroup sizes. As a result, part of the observed volume-outcome association may reflect greater procedural complexity and case mix at low-volume centers. These findings align with prior research, reinforcing the importance of centralizing SM care to high-volume centers while maintaining widespread access to TASH [13, 25]. Our study supports current guidelines advocating for SRT, particularly SM, to be performed at high-volume centers with specialized expertise [6, 10]. The higher mortality rates at low-volume centers highlight the risks associated with decentralization and emphasize the need for regional referral networks. For TASH, the findings reaffirm its role as an effective alternative for high-risk surgical candidates, provided careful patient selection and procedural planning are prioritized.

### Limitations

Several limitations should be acknowledged when interpreting this study. The use of anonymized administrative data restricts clinical granularity, such as echocardiographic findings, symptom severity, and long-term outcomes. ICD-10-GM and OPS coding may introduce inaccuracies in identifying procedures and diagnoses. Additionally, this study captures only in-hospital outcomes, leaving long-term survival, quality of life, and recurrence rates unexplored. Importantly, the symptomatic improvement and long-term outcomes was not compared in this study [26]. While our data show that more than one-third of SM procedures included concomitant mitral valve surgery, further differentiation between isolated SM and SM with other complex cardiac procedures (e.g., coronary artery bypass graft, valve replacement) was not feasible due to data protection requirements and low case numbers in several subgroups. Likewise, institutional volumes of other cardiac surgeries could not be reliably assessed. Therefore, the observed volume-outcome associations may partly reflect procedural complexity and surgical case mix. Lastly, the study period predates the introduction of pharmacological advancements like mavacamten, which may influence future treatment strategies by reducing the need for SRT in certain patients.

## Conclusion

In this nationwide cohort study, TASH was more commonly performed than SM, with both procedures increasing steadily over time. SM patients were older, had more comorbidities, and often required concomitant mitral valve surgery, reflecting greater procedural complexity. Nearly half of SM centers and over three-quarters of TASH centers were low-volume institutions, indicating limited centralization of SRT in Germany. Although low SM volumes were associated with higher in-hospital mortality, this may partly reflect unmeasured differences in procedural complexity and patient selection. TASH outcomes were unaffected by institutional volume. These findings underscore the need for individualized treatment strategies and centralization of SM at experienced centers to optimize outcomes in HOCM.

## Supplementary information

Below is the link to the electronic supplementary material.

Supplementary file 1 (DOCX 58.0 KB)

## Data Availability

The data underlying this article will be shared on reasonable request to the corresponding author.
