# Supplementary material for: Temporal trends, patient characteristics and hospital volume outcomes after transcoronary ablation of septal hypertrophy and septal myectomy for treatment of obstructive hypertrophic cardiomyopathy
Source: Clin Res Cardiol. 2025 Oct 9;115(5):842–51. doi: 10.1007/s00392-025-02773-7 (PMC13083484; doi:10.1007/s00392-025-02773-7)
Supplement: Supplementary file 1 — (DOCX 58.0 KB) [file 392_2025_2773_MOESM1_ESM.docx]

**Supplemental Material**

Supplemental Material to: Becher et al., Temporal Trends, Patient Characteristics and Hospital Volume Outcomes after Transcoronary Ablation of Septal Hypertrophy and Septal Myectomy for Treatment of Obstructive Hypertrophic Cardiomyopathy

**Table of Contents**  **Page number**

**Supplemental Tables**

**Supplemental Table S1. 2**

Temporal Trends in Baseline Characteristics, Treatments, and

Outcomes of Patients Underdoing Transcoronary Ablation of Septal Hypertrophy

**Supplemental Table S2. 5**

Temporal Trends in Baseline Characteristics, Treatments, and

Outcomes of Patients Underdoing Septal Myectomy

**Supplemental Table S3. 8**

Baseline Characteristics, Treatments, and Outcomes of Patients

According to Transcoronary Ablation of Septal Hypertrophy Volume Tertiles

**Supplemental Table S4. 9**

Baseline Characteristics, Treatments, and Outcomes of Patients

According to Septal Myectomy Volume Tertiles

**Supplemental Table S1.** Temporal Trends in Baseline Characteristics, Treatments, and Outcomes of Patients Underdoing Transcoronary Ablation of Septal Hypertrophy

| **Variables** | **2006 (n=329)** | **2007 (n=353)** | | **2008 (n=336)** | **2009 (n=361)** | **2010 (n=371)** | **2011**  **(n=350)** | **2012**  **(n=360)** | **2013**  **(n=377)** | **2014**  **(n=359)** | **2015**  **(n=347)** | **2016**  **(n=480)** | **2017**  **(n=381)** | **2018**  **(n=438)** | **2019**  **(n=451)** | **p-value** |
| --- | --- | --- | --- | --- | --- | --- | --- | --- | --- | --- | --- | --- | --- | --- | --- | --- |
| **Demographics** |  |  | |  |  |  |  |  |  |  |  |  |  |  |  |  |
| Age (years, SD) | 58.6 ± 14.2 | 59.8 ± 14.5 | | 59.6 ± 15.0 | 58.2 ± 15.1 | 58.5 ± 14.6 | 59.4 ± 14.0 | 61.6 ± 12.7 | 60.4 ± 12.7 | 60.9 ± 13.5 | 60.8 ± 13.7 | 59.8 ± 14.5 | 59.6 ± 14.3 | 61.8 ± 13.8 | 62.3 ± 13.1 | <0.001 |
| Female (%) | 177 (53.8%) | 172 (48.7%) | | 168 (50.0%) | 180 (49.8%) | 170 (45.8%) | 167 (47.7%) | 190 (52.7%) | 176 (46.6%) | 187 (52.0%) | 176 (50.7%) | 229 (47.7%) | 185 (48.5%) | 233 (53.2%) | 233 (51.6%) | 0.485 |
| Length of hospital stay (days) | 12.4 ± 10.3 | 11.7 ± 6.9 | | 11.5 ± 6.6 | 11.2 ± 99 | 10.6 ± 99 | 10.89 ± 99 | 11.01 ± 7.9 | 10.7 ± 7.1 | 10.0 ± 99 | 9.9 ± 6.2 | 10.1 ± 7.1 | 9.9 ± 10.0 | 9.6 ± 6.7 | 9.0  ± 99 | 1 |
| ***Comorbidities*** |  |  |  | |  |  |  |  |  |  |  |  |  |  |  |  |
| Arterial hypertension (%) | 90 (27.4%) | 102 (28.9%) | | 85 (25.3%) | 92 (25.4%) | 71 (19.1%) | 65 (18.5%) | 68 (18.8%) | 79 (20.9%) | 61 (16.9%) | 46 (13.2%) | 85 (17.7%) | 57 (14.9%) | 60 (13.7%) | 59 (13.1%) | <0.001 |
| Hyperlipoproteinemia (%) | 152 (46.2%) | 146 (41.3%) | | 114 (33.9%) | 129 (35.7%) | 128 (34.5%) | 125 (35.7) | 144 (40.0%) | 144 (38.2%) | 127 (35.3%) | 123 (35.4%) | 186 (38.7%) | 136 (35.7%) | 177 (40.4%) | 175 (38.8%) | 0.06 |
| Diabetes mellitus (%) | 42 (12.7%) | 34 (9.6%) | | 51 (15.1%) | 48 (13.3%) | 39 (10.5%) | 35 (10.0%) | 36 (10.0%) | 57 (15.1%) | 40 (11.1%) | 47 (13.5%) | 52 (10.8%) | 39 (10.2%) | 46 (10.5%) | 60 (13.3%) | 0.187 |
| Atrial fibrillation (%) | 47 (14.2%) | 52 (14.7%) | | 68 (20.2%) | 59 (16.3%) | 65 (17.5%) | 69 (19.7%) | 68 (18.8%) | 73 (19.3%) | 64 (17.8%) | 65 (18.7%) | 99 (20.6%) | 73 (19.1%) | 70 (15.9%) | 105 (23.2%) | 0.091 |
| Obesity (%) | 0  (0.0%) | 0  (0.0%) | | 39 (11.6%) | 44 (12.1%) | 48 (12.9%) | 47 (13.4%) | 44 (12.2%) | 56 (14.8%) | 63 (17.5%) | 60 (17.2%) | 76 (15.8%) | 64 (16.8%) | 58 (13.2%) | 50 (11.1%) | <0.001 |
| COPD (%) | na | na | | 0 (0.0%) | 0  (0.0%) | na | 0  (0.0%) | 0  (0.0%) | 0  (0.0%) | na | 0 (0.0%) | na | 0 (0.0%) | 0 (0.0%) | na |  |
| Anemia (%) | 11 (3.3%) | 18 (5.1%) | | 10 (2.9%) | 13 (3.6%) | 12 (3.2%) | 13 (3.7%) | 10 (2.7%) | 17 (4.5%) | 11 (3.0%) | 5 (1.4%) | 16 (3.3%) | 16 (4.2%) | 10 (2.2%) | 17 (3.7%) | 0.516 |
| Pulmonary hypertension (%) | 7  (2.1%) | 7  (1.9%) | | na | 3  (0.8%) | 5  (1.3%) | na | 11  (3.0%) | 9  (2.3%) | 11 (3.0%) | 9 (2.5%) | 11 (2.2%) | 7 (1.8%) | 12 (2.7%) | 6  (1.3%) |  |
| Chronic kidney disease (%) | 4  (1.2%) | 4  (1.1%) | | 4 (1.1%) | 8  (2.2%) | 50 (13.4%) | 49 (14.0%) | 35 (9.7%) | 45 (11.9%) | 44 (12.2%) | 36 (10.3%) | 52 (10.8%) | 44 (11.5%) | 57 (13.0%) | 56 (12.4%) | <0.001 |
| Heart failure (%) | 15 (4.5%) | 9  (2.5%) | | 16 (4.7%) | 14 (3.8%) | 11 (2.9%) | 14  (4.0%) | 16 (4.4%) | 5  (1.3%) | 21 (5.8%) | 21 (6.0%) | 13 (2.7%) | 14 (3.6%) | 19 (4.3%) | 15 (3.3%) | 0.067 |
| Stroke (%) | 4 (1.2%) | na | | na | na | na | na | na | 3  (0.8%) | 3  (0.8%) | 3 (0.8%) | na | na | 5 (1.1%) | 4  (0.8%) |  |
| Liver disease (%) | 3  (0.9%) | na | | na | 4  (1.1%) | na | na | na | 7  (1.8%) | 4  (1.1%) | na | 4  (0.8%) | 4 (1.0%) | 9 (2.0%) | 8  (1.7%) |  |
| Coagulopathy (%) | 42 (12.7%) | 33 (9.3%) | | 51 (15.1%) | 52 (14.4%) | 50 (13.4%) | 57 (16.2%) | 63 (17.5%) | 58 (15.3%) | 63 (17.5%) | 52 (14.9%) | 89 (18.5%) | 52 (13.6%) | 81 (18.4%) | 71 (15.7%) | 0.035 |
| Invasive ventilation (%) | 5  (1.5%) | 10  (2.8%) | | 7  (2.0%) | 6  (1.6%) | 3  (0.8%) | 5  (1.4%) | 4  (1.1%) | 7  (1.8%) | 4  (1.1%) | 8 (2.3%) | 5 (1.0%) | 6 (1.5%) | 14 (3.2%) | 8  (1.7%) | 0.367 |
| Non-invasive ventilation (%) | na | na | | na | na | na | na | 6  (1.6%) | 7  (1.8%) | 4  (1.1%) | na | 6 (1.2%) | 5 (1.3%) | 12 (2.7%) | 10 (2.2%) |  |
| Dialysis (%) | na | 3  (0.8%) | | na | na | na | na | na | 3  (0.8%) | na | na | 6 (1.2%) | na | 3 (0.6%) | na |  |
| History of MI (%) | 4 (1.2%) | 11 (3.1%) | | 6 (1.7%) | 7  (1.9%) | 5  (1.3 %) | 7  (2.0%) | 8  (2.2%) | 8  (2.1%) | 4  (1.1%) | 5 (1.4%) | 7 (1.4%) | 7 (1.8%) | 6 (1.3%) | 7 (1.5%) | 0.874 |
| History of CABG (%) | 0  (0.0%) | na | | na | 0  (0.0%) | 0  (0.ß%) | na | na | na | na | na | 3  (0.6%) | na | na | 0  (0.0%) |  |
| PAD (%) | 102 (31.0%) | 97 (27.4%) | | 104 (30.9%) | 105 (29.0%) | 141 (38.0%) | 124 (35.4%) | 157 (43.6%) | 164 (43.5%) | 149 (41.5%) | 145 (41.7%) | 204 (42.5%) | 162 (42.5%) | 226 (51.6%) | 208 (46.1%) | <0.001 |
| Coronary intervention (%) | 329 (100%) | 353 (100%) | | 336 (100%) | 361 (100%) | 371 (100%) | 350 (100%) | 360 (100%) | 377 (100%) | 359 (100%) | 347 (100%) | 480 (100%) | 381 (100%) | 438 (100%) | 451 (100%) |  |
| Coronary angiogram (%) | 297 (90.2%) | 315 (89.2%) | | 285 (84.8%) | 324 (89.7%) | 324 (87.3%) | 307 (87.7%) | 314 (87.2%) | 334 (88.5%) | 312 (86.9%) | 299 (86.1%) | 405 (84.3%) | 329 (86.3%) | 371 (84.7%) | 385 (85.3%) | 0.258 |
| Mitral valve surgery (%) | na | 0  (0.0%) | | 0 (0.0%) | 0  (0.0%) | 0  (0.0%) | na | 0  (0.0%) | 0  (0.0%) | 0  (0.0%) | 0 (0.0%) | na | 0 (0.0%) | na | 0  (0.0%) |  |
| ***Outcomes*** |  |  | |  |  |  |  |  |  |  |  |  |  |  |  |  |
| Acute renal failure (%) | na | na | | 0 (0.0%) | 0  (0.0%) | 0  (0.0%) | 0  (0.0%) | 6  (1.6%) | 6  (1.5%) | 6  (1.6%) | 12 (3.4%) | 16 (3.3%) | 19 (4.9%) | 4  (0.9 %) | 3  (0.6%) |  |
| Bleeding/transfusion (%) | 13 (3.9%) | 16 (4.5%) | | 12 (3.5%) | 13 (3.6%) | 9 (2.4%) | 11 (3.1%) | 13 (3.6%) | 17 (4.5%) | 13 (3.6%) | 5 (1.4%) | 17 (3.5%) | 13 (3.4%) | 13 (2.9%) | 18 (3.9%) | 0.771 |
| Mechanical circulatory  support (%) | na | na | | na | na | 0  (0.0%) | 0  (0.0%) | 0  (0.0%) | 3  (0.8%) | 0  (0.0%) | 0 (0.0%) | na | na | na | 3  (0.6%) |  |
| Pacemaker implantation (%) | 55 (16.7%) | 61 (17.2%) | | 70 (20.8%) | 66 (18.2%) | 68 (18.3%) | 65 (18.5%) | 90 (25.0%) | 80 (21.2%) | 65 (18.1%) | 58 (16.7%) | 87 (18.1%) | 74 (19.4%) | 103 (23.5%) | 79 (17.5%) | 0.095 |
| In-hospital mortality (%) | 6  (1.8%) | 4  (1.1%) | | na | na | 3  (0.8%) | na | 0  (0.0%) | 6  (1.5%) | 3  (0.8%) | na | 5 (1.0%) | 3 (0.7%) | na | na |  |

**Abbreviations:** coronary artery bypass graft (CABG), chronic obstructive pulmonary disease (COPD), myocardial infarction (MI), not available (na), peripheral artery disease (PAD), standard deviation (SD).

**Supplemental Table S2.** Temporal Trends in Baseline Characteristics, Treatments, and Outcomes of Patients Underdoing Septal Myectomy

| **Variables** | **2006 (n=191)** | **2007 (n=172)** | | **2008 (n=183)** | **2009 (n=180)** | **2010 (n=179)** | **2011**  **(n=265)** | **2012**  **(n=265)** | **2013**  **(n=322)** | **2014**  **(n=297)** | **2015**  **(n=278)** | **2016**  **(n=226)** | **2017**  **(n=222)** | **2018**  **(n=221)** | **2019**  **(n=220)** | **p-value** |
| --- | --- | --- | --- | --- | --- | --- | --- | --- | --- | --- | --- | --- | --- | --- | --- | --- |
| **Demographics** |  |  | |  |  |  |  |  |  |  |  |  |  |  |  |  |
| Age (years, SD) | 68.3 ± 12.9 | 70.0 ± 10.7 | | 66.5 ± 14.1 | 66.8 ± 13.6 | 66.9 ± 13.4 | 68.1 ± 12.1 | 68.6 ± 11.9 | 68.8 ± 11.7 | 68.0 ± 13.1 | 66.9 ± 13.0 | 66.5 ± 12.6 | 65.7 ± 13.1 | 65.7 ± 12.7 | 65.6 ± 11.3 | 0.003 |
| Female (%) | 119 (62.3%) | 106 (61.6%) | | 113 (61.7%) | 93 (51.6%) | 111 (62.0%) | 153 (57.7%) | 157 (59.2%) | 174 (54.0%) | 170 (57.2%) | 142 (51.0%) | 131 (57.9%) | 134 (60.3%) | 114 (51.5%) | 101 (45.9%) | 0.006 |
| Length of hospital stay (days) | 22.8 ± 19.7 | 19.6 ± 16.3 | | 19.2 ± 14.2 | 19.2 ± 13.0 | 17.4 ± 11.2 | 17.7 ± 12.6 | 16.7 ± 10.7 | 16.7 ± 9.5 | 17.4 ± 13.8 | 17.2 ± 10.8 | 20.1 ± 19.1 | 19.0 ± 14.9 | 17.5 ± 19.2 | 16.2 ± 11.0 | <0.001 |
| ***Comorbidities*** |  |  |  | |  |  |  |  |  |  |  |  |  |  |  |  |
| Arterial hypertension (%) | 30 (15.7%) | 20 (11.6%) | | 24 (13.1%) | 25 (13.8%) | 35 (19.5%) | 57 (21.5%) | 38 (14.3%) | 74 (22.9%) | 68 (22.9%) | 44 (15.8%) | 30 (13.2%) | 33 (14.8%) | 34 (15.3%) | 39 (17.7%) | 0.002 |
| Hyperlipoproteinemia (%) | 64 (33.5%) | 56 (32.5%) | | 80 (43.7%) | 67 (37.2%) | 65 (36.3%) | 123 (46.4%) | 130 (49.0%) | 141 (43.7%) | 128 (43.%) | 111 (39.9%) | 97 (42.9%) | 105 (47.%) | 100 (45.2%) | 106 (48.1%) | 0.003 |
| Diabetes mellitus (%) | 45 (23.5%) | 31 (18.0%) | | 33 (18.0%) | 36 (20.0%) | 28 (15.6%) | 52 (19.6%) | 55 (20.7%) | 48 (14.9%) | 67 (22.5%) | 42 (15.1%) | 46 (20.3%) | 39 (17.5%) | 47 (21.2%) | 28 (12.7%) | 0.086 |
| Atrial fibrillation (%) | 84 (43.9%) | 83 (48.2%) | | 85 (46.4%) | 72 (40.0%) | 71 (39.6%) | 119 (44.9%) | 129 (48.6%) | 151 (46.8%) | 153 (51.5%) | 128 (46.0%) | 123 (54.4%) | 103 (46.4%) | 92 (41.6%) | 92 (41.8%) | 0.083 |
| Obesity (%) | 0 (0.0%) | na | | 42 (22.9%) | 37 (20.5%) | 47 (26.2%) | 55 (20.7%) | 47 (17.7%) | 56 (17.3%) | 52 (17.5%) | 60 (21.5%) | 51 (22.5%) | 38 (17.1%) | 52 (23.5%) | 37 (16.8%) |  |
| COPD (%) | 3  (1.5%) | 7  (4.0%) | | 5 (2.7%) | 5  (2.7%) | na | 9 (3.4%) | 3 (1.1%) | 8 (2.4%) | na | 5 (1.8%) | na | na | na | 0 (0.0%) |  |
| Anemia (%) | 110 (57.5%) | 120 (69.7%) | | 104 (56.8%) | 113 (62.7%) | 130 (72.6%) | 185 (69.8%) | 200 (75.4%) | 231 (71.7%) | 209 (70.3%) | 197 (70.8%) | 160 (70.%) | 144 (64.8%) | 148 (66.9%) | 143 (65.0%) | <0.001 |
| Pulmonary hypertension (%) | 3 (1.5%) | 3 (1.7%) | | 0 (0.0%) | 4  (2.2%) | na | 3 (1.1%) | 9 (3.4%) | 6 (1.8%) | 4 (1.3%) | 7 (2.5%) | na | 8 (3.6%) | 3 (1.3%) | 4 (1.8%) |  |
| Chronic kidney disease (%) | 6 (3.1%) | 6 (3.4%) | | 6 (3.2%) | 8  (4.4%) | 31 (17.3%) | 53 (20.0%) | 57 (21.5%) | 49 (15.2%) | 51 (17.1%) | 46 (16.5%) | 41 (18.1%) | 46 (20.7%) | 31 (14.0%) | 31 (14.0%) | <0.001 |
| Heart failure (%) | 20 (10.4%) | 18 (10.4%) | | 14 (7.6%) | 17  (9.4%) | 17 (9.5%) | 35 (13.2%) | 46 (17.3%) | 28 (8.7%) | 37 (12.4%) | 36 (12.9%) | 31 (13.7%) | 33 (14.8%) | 30 (13.5%) | 38 (17.2%) | 0.024 |
| Stroke (%) | 20 (10.4%) | 16 (9.3%) | | 16 (8.7%) | 15 (8.3%) | 21 (11.7%) | 22 (8.3%) | 16 (6.0%) | 31 (9.6%) | 32 (10.7%) | 24 (8.6%) | 16 (7.0%) | 22 (9.9%) | 20 (9.0%) | 18 (8.1%) | 0.84 |
| Liver disease (%) | 7  (3.6%) | 6  (3.4%) | | 9 (4.9%) | 4  (2.2%) | 9  (5.0%) | 20 (7.5%) | 14 (5.2%) | 7  (2.1%) | 14 (4.7%) | 20 (7.1%) | 18 (7.9%) | 13 (5.8%) | 19 (8.1%) | 15 (6.8%) | 0.022 |
| Coagulopathy (%) | 59 (30.8%) | 64 (37.2%) | | 60 (32.7) | 58 (32.2%) | 72 (40.2%) | 99 (37.3%) | 114 (43.0%) | 156 (48.4%) | 169 (56.%) | 160 (57.5%) | 123 (54.4%) | 121 (54.5%) | 125 (56.5) | 126 (57.2%) | <0.001 |
| Invasive ventilation (%) | 59 (30.8%) | 41 (23.8%) | | 44 (24.0) | 45 (25.0%) | 34 (18.9%) | 70 (26.4%) | 52 (19.6%) | 82 (25.4%) | 88 (29.6%) | 90 (32.3%) | 70 (30.9%) | 56 (25.2%) | 42 (19.0) | 44 (20.0%) | 0.001 |
| Non-invasive ventilation (%) | 6  (3.1%) | 8  (4.6%) | | 7 (3.8%) | 4  (2.2%) | 14 (7.8%) | 20 (7.5%) | 25 (9.4%) | 41 (12.7%) | 51 (17.1%) | 43 (15.4%) | 26 (11.5%) | 40 (18.0%) | 32 (14.4) | 29 (13.1%) | <0.001 |
| Dialysis (%) | 44 (23.0%) | 38 (22.0%) | | 50 (27.3%) | 29 (16.1%) | 24 (13.4%) | 43 (16.2%) | 34 (12.8%) | 27 (8.3%) | 35 (11.7%) | 28 (10.0%) | 25 (11.0%) | 18 (8.1%) | 14 (6.3%) | 19 (8.6%) | <0.001 |
| History of MI (%) | 9  (4.7%) | 4  (2.3%) | | 11 (6.0%) | 5  (2.7%) | 9  (5.0%) | 15 (5.6%) | 10 (3.7%) | 19 (5.9%) | 14 (4.7%) | 10 (3.6%) | 14 (6.1%) | 11 (4.9%) | 15 (6.7%) | 7 (3.1%) | 0.565 |
| History of CABG (%) | 8  (4.1%) | 10 (5.8%) | | 4 (2.1%) | 4  (2.2%) | 3  (1.6%) | 8  (3.0%) | 5  (1.8%) | 10 (3.1%) | 6  (2.0%) | na | na | 5 (2.2%) | 0 (0.0%) | 0 (0.0%) |  |
| PAD (%) | 94 (49.2%) | 77 (44.7%) | | 83 (45.3%) | 84 (46.6%) | 87 (48.6%) | 131 (49.4%) | 105 (39.6%) | 104 (32.3%) | 114 (38.3%) | 141 (50.7%) | 121 (53.5%) | 117 (52.7%) | 129 (58.3%) | 113 (51.3%) | <0.001 |
| Coronary intervention (%) | 0  (0.0%) | na | | na | na | na | na | 5  (1.8%) | 0  (0.0%) | na | na | 5 (2.2%) | na | 3 (1.3%) | 4  (1.8%) | 0 (0.0%) |
| Coronary angiogram (%) | 56 (29.3%) | 34 (19.7%) | | 40 (21.8%) | 44 (24.4%) | 39 (21.7%) | 59 (22.2%) | 59 (22.2%) | 85 (26.4%) | 69 (23.2%) | 62 (22.3%) | 60 (26.5%) | 63 (28.3%) | 39 (17.6%) | 39 (17.7%) | 0.09 |
| Mitral valve surgery (%) | 74 (38.7%) | 67 (38.9%) | | 65 (35.5%) | 63 (35.0%) | 62 (34.6%) | 91 (34.3%) | 87 (32.8%) | 114 (35.4 %) | 101 (34.0%) | 86 (30.9%) | 89 (39.3%) | 102 (45.9%) | 77 (34.8%) | 86 (39.0%) | 0.147 |
| ***Outcomes*** |  |  | |  |  |  |  |  |  |  |  |  |  |  |  |  |
| Acute renal failure (%) | 0  (0.0%) | na | | na | na | na | na | 7  (2.6%) | 3  (0.9%) | 6  (2.0%) | 8 (2.8%) | 4 (1.7%) | 4 (1.8%) | 5 (2.2%) | 3  (1.3%) |  |
| Bleeding/transfusion (%) | 134 (70.1%) | 133 (77.3%) | | 134 (73.2%) | 133 (73.8%) | 141 (78.7%) | 201 (75.8%) | 213 (80.3%) | 237 (73.6%) | 217 (73.0%) | 193 (69.4%) | 162 (71.6%) | 148 (66.6%) | 150 (67.8%) | 143 (65.0%) | 0.005 |
| Mechanical circulatory  support (%) | 11 (5.7%) | 8  (4.6%) | | 12 (6.5%) | 8  (4.4%) | 5  (2.7%) | 12 (4.5%) | 12 (4.5%) | 10 (3.1%) | 11 (3.7%) | 4 (1.4%) | 6 (2.6%) | 5 (2.2%) | na | 8  (3.6%) |  |
| Pacemaker implantation (%) | 19 (9.9%) | 21 (12.2%) | | 32 (17.4%) | 22 (12.2%) | 25 (13.9%) | 41 (15.4%) | 31  (11.7%) | 32 (9.9%) | 42 (14.1%) | 33 (11.8%) | 33 (14.6%) | 31 (13.9%) | 35 (15.8%) | 24 (10.9%) | 0.42 |
| In-hospital mortality (%) | 22 (11.5%) | 20 (11.6%) | | 14 (7.6%) | 10 (5.5%) | 15 (8.3%) | 24 (9.0%) | 19 (7.1%) | 11 (3.4%) | 24 (8.0%) | 21 (7.5%) | 9 (3.9%) | 10 (4.5%) | 13 (5.8%) | 9  (4.0%) | 0.004 |

**Abbreviations:** coronary artery bypass graft (CABG), chronic obstructive pulmonary disease (COPD), myocardial infarction (MI), not available (na), peripheral artery disease (PAD), standard deviation (SD).

**Supplemental Table S3.** Baseline Characteristics, Treatments, and Outcomes of

Patients According to Transcoronary Ablation of Septal Hypertrophy Volume Tertiles

| **Variables** | **Third tertile**  **(n=4848)** | **Second tertile**  **(n=399)** | **First tertile**  **(n=46)** | **p-value** |
| --- | --- | --- | --- | --- |
| **Demographics** |  |  |  |  |
| Age (years, SD) | 59.9 ± 14.1 | 62.5 ± 12.9 | 68.5 ± 12.3 | <0.001 |
| Female (%) | 2404 (49.5%) | 208 (52.1%) | 31 (67.3%) | 0.037 |
| Length of hospital stay (days) | 10.5 ± 6.5 | 10.8 ± 10.8 | 12.9 ± 11.0 | 0.049 |
| ***Comorbidities*** |  |  |  |  |
| Arterial hypertension (%) | 960 (19.8%) | 54 (13.5%) | 6 (13.1%) | 0.005 |
| Hyperlipoproteinemia (%) | 1835 (37.8%) | 152 (38.1%) | 19 (41.3%) | 0.888 |
| Diabetes mellitus (%) | 561 (11.5%) | 55 (13.7%) | 10 (21.7%) | 0.047 |
| Atrial fibrillation (%) | 868 (17.9%) | 91 (22.8%) | 18 (39.1%) | <0.001 |
| Obesity (%) | 596 (12.2%) | 50 (12.5%) | 3 (6.5%) | <0.001 |
| COPD (%) | 8 (0.2%) | na | 0 (0%) |  |
| Anemia (%) | 157 (3.2%) | 17 (4.2%) | 5 (10.8%) | 0.01 |
| Pulmonary hypertension (%) | 87 (1.7%) | 10 (2.5%) | 4 (8.7%) | 0.002 |
| Chronic kidney disease (%) | 431 (8.8%) | 48 (12.0%) | 9 (19.5%) | 0.006 |
| Heart failure (%) | 184 (3.8%) | 17 (4.2%) | na |  |
| Stroke (%) | 26 (0.5%) | 6 (1.5%) | na |  |
| Liver disease (%) | 46 (0.9%) | 7 (1.7%) | 0 (0%) | 0.237 |
| Coagulopathy (%) | 726 (14.9%) | 79 (19.8%) | 9 (19.5%) | 0.02 |
| Invasive ventilation (%) | 79 (1.6%) | 11 (2.7%) | na |  |
| Non-invasive ventilation (%) | 50 (1.0%) | 8 (2.0%) | 3 (6.5%) | 0.001 |
| Dialysis (%) | 28 (0.5%) | 4 (1.0%) | 0 (0%) | 0.499 |
| History of MI (%) | 80 (1.6%) | 9 (2.2%) | 3 (6.5%) | 0.03 |
| History of CABG (%) | 16 (0.3%) | na | 0 (0%) |  |
| PAD (%) | 1884 (38.8%) | 180 (45.1%) | 24 (52.1%) | 0.01 |
| Coronary intervention (%) | 4848 (100%) | 399 (100%) | 46 (100%) |  |
| Coronary angiogram (%) | 4230 (87.2%) | 333 (83.4%) | 38 (82.6%) | 0.066 |
| Mitral valve surgery (%) | 4 (0.1%) | 0 (0%) | 0 (0%) | 0.832 |
| ***Outcomes*** |  |  |  |  |
| Acute renal failure (%) | 70 (1.4%) | na | 3 (6.5%) |  |
| Bleeding/transfusion (%) | 160 (3.3%) | 18 (4.5%) | 5 (10.8%) | 0.01 |
| Mechanical circulatory  support (%) | 13 (0.2%) | na | 0 (0%) |  |
| Pacemaker implantation (%) | 944 (19.4%) | 70 (17.5%) | 7 (15.2%) | 0.503 |
| In-hospital mortality (%) | 38 (0.7%) | na | na |  |

**Abbreviations:** transcoronary ablation of septal hypertrophy (TASH), coronary artery bypass graft (CABG), chronic obstructive pulmonary disease (COPD), myocardial infarction (MI), not available (na), peripheral artery disease (PAD), standard deviation (SD).

**Supplemental Table S4.** Baseline Characteristics, Treatments, and Outcomes of Patients According to Septal Myektomy Volume Tertiles

| **Variables** | **Third tertile**  **(n=2487)** | **Second tertile**  **(n=567)** | **First tertile**  **(n=167)** | **p-value** |
| --- | --- | --- | --- | --- |
| **Demographics** |  |  |  |  |
| Age (years, SD) | 67.8 ± 12.3 | 65.5 ± 13.9 | 67.5 ± 11.9 | <0.001 |
| Female (%) | 1399 (56.2%) | 316 (55.7%) | 103 (61.6%) | 0.365 |
| Length of hospital stay (days) | 17.9 ± 13.6 | 18.1 ± 13.3 | 21.8 ± 22.7 | 0.003 |
| ***Comorbidities*** |  |  |  |  |
| Arterial hypertension (%) | 430 (17.2%) | 88 (15.5%) | 33 (19.7%) | 0.388 |
| Hyperlipoproteinemia (%) | 1099 (44.1%) | 207 (36.5%) | 67 (40.1%) | 0.003 |
| Diabetes mellitus (%) | 470 (18.9%) | 100 (17.6%) | 27 (16.1%) | 0.565 |
| Atrial fibrillation (%) | 1173 (47.1%) | 229 (40.3%) | 83 (49.7%) | 0.009 |
| Obesity (%) | 462 (18.5%) | 85 (14.9%) | 28 (16.7%) | 0.123 |
| COPD (%) | 43 (1.7%) | 4 (0.7%) | 5 (2.9%) | 0.076 |
| Anemia (%) | 1718 (69.0%) | 367 (64.7%) | 109 (65.2%) | 0.096 |
| Pulmonary hypertension (%) | 45 (1.8%) | 7 (1.2%) | 4 (2.4%) | 0.512 |
| Chronic kidney disease (%) | 364 (14.6%) | 80 (14.1%) | 18 (10.7%) | 0.382 |
| Heart failure (%) | 331 (13.3%) | 53 (9.3%) | 16 (9.5%) | 0.019 |
| Stroke (%) | 239 (9.6%) | 38 (6.7%) | 12 (7.1%) | 0.065 |
| Liver disease (%) | 130 (5.2%) | 35 (6.1%) | 10 (5.9%) | 0.635 |
| Coagulopathy (%) | 1166 (46.8%) | 273 (48.1%) | 67 (40.1%) | 0.182 |
| Invasive ventilation (%) | 632 (25.4%) | 136 (23.9%) | 49 (29.3%) | 0.374 |
| Non-invasive ventilation (%) | 272 (10.9%) | 50 (8.8%) | 24 (14.3%) | 0.101 |
| Dialysis (%) | 361 (14.5%) | 50 (8.8%) | 17 (10.1%) | 0.001 |
| History of MI (%) | 123 (4.9%) | 20 (3.5%) | 10 (5.9%) | 0.266 |
| History of CABG (%) | 58 (2.3%) | na | 5 (2.9%) |  |
| PAD (%) | 1165 (46.8%) | 267 (47.0%) | 68 (40.7%) | 0.296 |
| Coronary intervention (%) | 22 (0.8%) | na | na |  |
| Coronary angiogram (%) | 557 (22.4%) | 152 (26.8%) | 39 (23.3%) | 0.08 |
| Mitral valve surgery (%) | 917 (36.8%) | 199 (35.1%) | 48 (28.7%) | 0.091 |
| ***Outcomes*** |  |  |  |  |
| Acute renal failure (%) | 32 (1.2%) | 13 (2.2%) | na |  |
| Bleeding/transfusion (%) | 1815 (72.9%) | 402 (70.9%) | 122 (73.0%) | 0.6 |
| Mechanical circulatory  support (%) | 100 (4.0%) | 11 (1.9%) | 3 (1.8%) | 0.024 |
| Pacemaker implantation (%) | 318 (12.7%) | 78 (13.7%) | 25 (14.9%) | 0.624 |
| In-hospital mortality (%) | 157 (6.3%) | 46 (8.11 %) | 18 (10.7%) | 0.037 |

**Abbreviations:** coronary artery bypass graft (CABG), chronic obstructive pulmonary disease (COPD), myocardial infarction (MI), not available (na), peripheral artery disease (PAD), standard deviation (SD), surgical myectomy (SM).
